# Digital health, gender and health equity: invisible imperatives

**DOI:** 10.1093/pubmed/fdy171

**Published:** 2018-10-17

**Authors:** Chaitali Sinha, Anne-Marie Schryer-Roy

**Affiliations:** 1 International Development Research Centre (IDRC), Ottawa, Canada; 2 Nairobi, Kenya

**Keywords:** m-Health, mHealth, eHealth, digital health, gender, health equity, women, SDGs, accountability, health systems

## Abstract

A growing body of evidence shows the use of digital technologies in health—referred to as eHealth, mHealth or ‘digital health’—is improving and saving lives in low- and middle-income countries. Despite this prevalent and persistent narrative, very few studies examine its effects on health equity, gender and power dynamics. This journal supplement addresses these invisible imperatives by going beyond traditional measures of coverage, efficacy and cost-effectiveness associated with digital health interventions, to unpack different experiences of health workers and beneficiaries. The collection of papers presents findings from a cohort of implementation research projects in Africa, Asia, Latin America and the Middle East, and two commentaries offer observations from learning-oriented evaluative activities across the entire cohort. The story emerging from this cohort is comprised of three themes: (i) digital health can positively influence health equity; (ii) gender and power analyses are essential; and (iii) digital health can be used to strengthen upward and downward accountability. These findings, at the individual project level and at the level of the cohort, provide encouraging recommendations on how to approach the design, implementation and evaluation of digital health interventions to address the Sustainable Development Goals agenda of leaving no one behind.

## RESUMEN

Una cantidad creciente de evidencia muestra que el uso de las tecnologías digitales en la salud, ya sea eSalud (eHealth), mSalud (mHealth) o "salud digital", está mejorando y salvando vidas en países de ingresos bajos y medios. A pesar de esta descripción frecuente y persistente, muy pocos estudios analizan sus efectos en la igualdad en materia de salud y las dinámicas de género y de poder. Este suplemento informativo aborda estos imperativos invisibles al ir más allá de las medidas de cobertura tradicionales, la eficiencia y la eficacia en función de los costos asociadas con las intervenciones de salud digital, para desentrañar las diferentes experiencias de los beneficiarios y trabajadores de la salud. Este conjunto de trabajos presenta resultados de una cohorte de aplicación de proyectos de investigación en África, Asia, América Latina y el Medio Oriente; y dos comentarios ofrecen observaciones de actividades de evaluación orientadas en el aprendizaje en toda la cohorte. La historia que surge de esta cohorte comprende tres temas: (i) la salud digital puede influenciar positivamente la igualdad en materia de salud; (ii) los análisis de género y de poder son esenciales; y (iii) la salud digital puede usarse para fortalecer la rendición de cuentas ascendente y descendente. Estos resultados, a nivel de proyecto individual y al nivel de la cohorte, brindan recomendaciones alentadoras acerca de cómo abordar el diseño, la aplicación y la evaluación de las intervenciones de salud digital para examinar la agenda de los Objetivos de Desarrollo Sostenible de no dejar a nadie atrás.

**Palabras clave:** m-Salud, mSalud, eSalud, salud digital, género, igualdad en materia de salud, mujeres, ODS, rendición de cuentas, sistemas de salud

## RÉSUMÉ

De plus en plus d’études révèlent que le recours aux technologies numériques en santé – désignées par les termes cybersanté, santé mobile ou santé numérique – permet de sauver des vies dans les pays à revenu faible ou moyen et d’en améliorer la qualité. Malgré la prévalence et la constance des données, très peu d’études traitent de ses répercussions sur l’équité en santé, la sexospécificité et la dynamique de pouvoir. Le présent supplément se penche sur ces impératifs invisibles et va au-delà des mesures traditionnelles de couverture, d’efficacité et de rentabilité associées aux interventions numériques en santé, afin de lever le voile sur les différentes expériences que vivent les travailleurs de la santé et les bénéficiaires de soins de santé. Ce recueil de textes présente les résultats d’une cohorte de projets de recherche sur la mise en oeuvre menés en Afrique, en Asie, en Amérique latine et au Moyen-Orient, et deux commentaires font part d’observations tirées d’activités d’évaluation axées sur l’apprentissage réalisées dans toute la cohorte. L’analyse des résultats de la cohorte aboutit à trois constats : (i) la santé numérique peut exercer une influence positive sur l’équité en santé; (ii) l’analyse comparative entre les sexospécificités et l’analyse de la dynamique de pouvoir sont essentielles; (iii) la santé numérique peut être utilisée pour renforcer la responsabilisation vers le haut et vers le bas. Ces constats, qu’ils soient liés à chacun des projets ou à l’ensemble de la cohorte, donnent lieu à des recommandations encourageantes quant à la manière d’aborder la conception, la mise en oeuvre et l’évaluation d’interventions numériques en santé, de sorte à réaliser le programme et les objectifs de développement durable consistant à ne laisser personne de côté.

**Mots clés:** santé mobile, cybersanté, santé numérique, sexospécificité, équité en santé, femmes, ODD, responsabilisation, systèmes de santé

## Introduction

The spread and uptake of digital health (including eHealth and mHealth) in low- and middle-income countries (LMICs) presents promising opportunities to extend coverage of, and access to, life-saving health services, customize health information for marginalized and vulnerable groups and improve upward and downward accountability of the health system.^1–4^ These opportunities directly contribute to achieving the Sustainable Development Goals (SDGs) and the commitment to ‘leaving no one behind’. If realized, these promises ensure healthy and productive lives for all women and children, central to achieving the Global Strategy for Women’s, Children’s and Adolescents’ Health.

But digital health in its engagement with complex social settings can have diverse impacts—positive, negative and mixed—on equitable health services and gender relations in communities. As a result, the absence of a strong health equity and gender analysis when designing, implementing and evaluating digital health policies and programmes can lead to ignoring or exacerbating existing health inequities and gender inequalities, or even creating new ones. Negotiating and navigating these norms and values to ensure digital health initiatives address local systems, processes and realities is an invisible imperative of fundamental importance.

There is a growing body of evidence on the use of digital health in LMICs, including systematic reviews on mHealth,^5^ and on maternal, newborn and child health.^6–8^ The literature has an increasing number of contributions on suitable digital health frameworks,^9^ and guidelines on how to report evidence.^10^ Questions of adoption, uptake, efficacy and changes in outcomes have also been answered to varying extents.^3^ Yet, there continues to be a dearth of studies that focus on health equity and gender as critical axes for analysis and action.^11,12^

This collection of seven original papers and two commentaries seeks to contribute to filling this gap in knowledge by presenting practical lessons and findings from a cohort of seven implementation research projects. Funded by Canada’s International Development Research Centre (IDRC), the projects were supported in Bangladesh, Burkina Faso, Ethiopia, Lebanon, Kenya, Peru and Vietnam from 2013 to 2017.

The framing of this cohort benefited from over 15 years of research for development programming by IDRC on digital health^13^ and a consultation with ‘thought leaders’ mostly from Africa, Asia and Latin America. The two commentaries share a cross-cohort perspective on gender analysis, and reflections from a developmental evaluation study on the cohort itself.

The story emerging from this collection is that the true potential of digital health can be harnessed only when we move beyond coverage statistics and apply systematic and periodic health equity and gender analysis towards targeted services and transformative change. A number of common themes within this narrative can be found across the papers. Although presented as standalone themes, the reader will detect some natural overlap and intentional reinforcement across the themes. Novel attributes of this collection of papers include: (i) a consistent and underlying emphasis on health inequity as the research for development problematique; (ii) a focus on rights-based intersectional gender analysis; and (iii) and a set of lessons emerging from supporting an integrated cohort of research projects across four regions (Africa, Asia, Latin America and the Middle East).

## Theme 1: Digital health can positively influence health equity

Focusing on health equity in the SDG era is a non-negotiable if we are, as a global community, striving to leave no one behind. The challenge now is finding the balance of theoretical grounding and grounded intervention to reduce health inequities.^14^ Unpacking social and political factors that affect health inequities is critical to addressing their root causes.^15^ Articles in this supplement focus on diverse marginalized groups across the globe such as ethnic minority women in Vietnam, refugee populations in Lebanon, indigenous women in Peru, people living with HIV in Burkina Faso and rural populations in Ethiopia. Several papers show that, under the right set of circumstances and with thoughtful design and implementation, digital health interventions can have a positive impact on health equity, as prefaced below.

In Burkina Faso, Yé *et al.*^16^ evaluated the performance of an mhealth intervention targeting pregnant women, new mothers and people living with HIV (PLHIV). It addressed health inequities evidenced by low treatment compliance rates and high loss to follow-up for PLHIV, as well as persistently high maternal and child mortality among the general population. The authors report an increase in prenatal coverage and assisted deliveries, and a decrease in the rate of loss to follow-up for PLHIV.

In Vietnam, McBride *et al.*^17^ piloted a low-cost mhealth intervention among ethnic minority women who experience disproportionately high incidence of infant and maternal mortality and suffer from other forms of exclusion. The researchers addressed local language needs and provided information about maternal and child health via SMS and their community healthcare workers. Results showed a measurable increase in demand for quality natal care. Findings also point to an increase in women’s confidence in accessing care, and greater supportive involvement of their husbands. These social transformations represent a critical step in overcoming marginalization and deprivation experienced by this group.

## Theme 2: Gender and power analyses are essential

Digital health interventions are often being implemented in contexts where gender inequalities, biases, class disparity and uneven power dynamics prevail.^18^ Gender analysis that considers power relations within and among females and males can be enriched by an intersectional perspective, which involves examining gender in relation to other social stratifiers, such as class, race, education, ethnicity, age, geographic location, (dis)ability and sexuality.^19^ Papers in this collection present approaches sensitive to existing social fabrics, tensions and needs, with findings that can contribute towards positively transforming gender norms and power dynamics.

In Ethiopia, Steege *et al.*^20^ examine the impact of an mHealth intervention on the gendered experiences of Health Extension Workers (HEWs) and how gender inequalities can weaken attempts to positively scale-up mHealth initiatives. The authors find that while the intervention enhanced HEWs social status and agency and allowed them to provide more equitable care to communities, the intervention had the potential to place an additional burden of work on an already overstretched cadre of healthcare workers.

Exploring information needs of pregnant indigenous women Peru, Pérez-Lu *et al.*^21^ found that providing them with access to their health records strengthened power, ownership and agency. The study also measured results related to the environment and the channel through which information was received—through digital and other means. A friendly and secure environment with supportive health workers—adopting a positive attitude and appropriate language—was shown to influence behaviour positively. These findings influenced how the mHealth innovation was designed and deployed.

## Theme 3: Digital health strengthening upward and downward accountability

The potential for digital health to enhance accountability among and between community members, community and facility-based health workers, supervisors and the health system was identified by several authors.^22^ Consistent across these experiences is the engagement with local communities and decision-makers to strengthen health governance.

In Lebanon, Saleh *et al.*^23^ explore both the demand and the supply side of providing much-needed non-communicable diseases health services to refugee and rural populations. Assessing the perceived quality and effectiveness of weekly SMS messages and reminders for appointments, the study found high rates of satisfaction overall, with some populations requiring more attention such as the elderly, non-literate and unemployed individuals.

In a second paper from rural Ethiopia, Mengesha *et al.*^24^ found that an mHealth solution, designed in an iterative and participatory way, enhanced bidirectional accountability at various levels, and between different actors. Focused on supporting the critical health provider groups, HEWs, the system aimed to improve tuberculosis and maternal health outcomes. Its dual focus on accountability of HEWs to the community, as well as strengthening bidirectional accountability between HEWs and their supervisors, provides much-needed findings that link improvements in health outcomes with how health workers are incentivized and supported.

## Concluding comments

Few digital health projects comprise health equity and gender analysis in their methodologies. Fewer still are supported in a cohort that is designed with intentional opportunities to share experiences and lessons, with some axes of analyses cutting across all projects. Decosas and Mbuagbaw^25^ discuss reflections and lessons from their journey accompanying the cohort through a real-time developmental learning evaluation. The evaluation included five areas of inquiry—analysis of cross-cutting concepts such as ‘health equity’, gender analysis, knowledge translation practices and outcomes, research quality and cross-cohort networking. Through this effort, gender analysis was identified as a shared area of interest among the research teams and one that could benefit from additional targeted capacity strengthening efforts. In their paper about gender in the cohort, George *et al.*^26^ provide an insightful overview of gender equality analysis in digital health, with specific lessons shared from the projects in this cohort.

The evidence that emerges from the articles presented in this supplement is encouraging, as many authors demonstrate how health equity and upward and downward accountability can be strengthened through well-designed digital health interventions. In that respect, these results confirm that digital health can indeed contribute to the goal of leaving no one behind. At the same time, the articles also highlight the need to pay attention to existing gender and power dynamics, and to take these into account when designing, implementing and evaluating any digital health intervention. Failure to do so could help to maintain, or even worsen, the status quo of underserved, marginalized populations.

As LMICs increasingly move forward with digital health innovations as part of their health systems, this supplement offers some food for thought and lessons from the field that can be adapted to different contexts. Above all, what the papers demonstrate is the need to integrate in their design a critical analysis of context-specific gender and power dynamics as a foundational requirement for any intervention.
